# Methodological issues in estimating survival in patients with multiple primary cancers: an application to women with breast cancer as a first tumour

**DOI:** 10.1186/1742-7622-6-2

**Published:** 2009-02-27

**Authors:** Stefano Rosso, Fulvio Ricceri, Lea Terracini, Roberto Zanetti

**Affiliations:** 1 Piedmont Cancer Registry – CPO, Turin, Italy; 2 Unit of Epidemiology and Modelling, Institute for Scientific Interchange (ISI) Foundation, Turin, Italy; 3 Department of Genetics, Biology and Biochemistry, University of Turin, Turin, Italy; 4 Department of Mathematics, University of Turin, Turin, Italy

## Abstract

**Background:**

Comparing survival of patients with a single tumour and patients with multiple primaries poses different methodological problems. In population based studies, where we cannot rely on detailed clinical information, the issue is disentangling the share of survival probability from the first and second cancer, and their compounded effect. We examined three hypotheses: A) the survival probability since the first tumour does not change with the occurrence of a second tumour; B) the probability of surviving a tumour does not change with the presence of a previous primary; C) the probabilities of surviving two subsequent primary tumours are independent (additivity hypothesis on mortality rates).

**Methods:**

We studied the survival probabilities modelling mortality rates according to hypotheses A), B) and C). Mortality rates were calculated using Aalen-Johansen estimators which allowed to discount for the lag-time survival before developing a second tumour. We applied this approach to a cohort of 436 women with breast cancer (BC) and a subsequent tumour in the resident population of Turin, Italy, between 1985 and 2002.

**Results:**

We presented our results in term of a Standardised Mortality Ratio calculated (*SMR*_*AJ*_) after 10 years of follow-up. For hypothesis A we observed a significant excess mortality of 2.21 (95% C.I. 1.94 – 2.45). Concerning hypothesis B we found a not significant *SMR*_*AJ *_of 0.98 (95% C.I. 0.87 – 1.10). The additivity hypothesis (C) was not confirmed as it overestimated the risk of death, in fact *SMRs*_*AJ *_were all below 1: 0.75 (95% C.I. 0.66 – 0.84) for BC and all subsequent cancers, 0.72 (95% C.I. 0.55 – 0.94) for BC and colon-rectum cancer, 0.76 (95% C.I. 0.48 – 1.14) for BC and corpus uteri cancer (not significant).

**Conclusion:**

This method proved to be useful in disentangling the effect of different subsequent cancers on mortality. In our application it shows a worse long-term mortality for women with two cancers than that with BC only. However, the increase in mortality was lower than expected under the additivity assumption.

## Introduction

The improvement of patients survival for the vast majority of neoplasms led to a substantial increase in the probability of further developing subsequent primary tumours. However, the study of multiple primary tumours on a population basis posed many additional problems. There is, indeed, a problem of differential diagnosis, when it comes to distinguish between local and distant metastases, recurrences and the onset of a truly new lesion. Classifications may also vary leading to substantial differences in rates. For example Surveillance Epidemiology and End Results (SEER) rules [1] differ substantially from those adopted by International Agency for Research on Cancer (IARC) [2].

Furthermore, survival of patients with multiple tumours has been neglected in population-based analyses, where they are usually list-wise deleted, or analysed for the first tumour occurrence only [3,4]. Only recently two studies [5,6] reconsidered this exclusion policy. On the contrary, in clinical series survival of patients with multiple tumours is usually defined clinically and specific cause of death is assessed accordingly. However, in population studies and in series from cancer registries, clinical information on patients follow-up is often unavailable and assessment of cause of death is based only on death certificates, often liable to gross misclassification. Heinävaara et al. [7] proposed to estimate the differential amount due to first or second tumour with a statistical parametric model. Their application dealt with patients with two primary breast cancers, where the question of disentangling the cancer-specific survival due to the first or the second tumour is more difficult, also from a clinical point of view. In the case of a subsequent primary cancer of a different origin the question is apparently simpler, although not yet investigated on a population basis.

The following questions can be raised: whether the overall survival of patients has decreased because of the interaction between the two cancers, or if it has been left substantially unchanged in comparison to those with one cancer only, or even increased. For example, active surveillance and care due to the first cancer can lead to earlier diagnosis of subsequent cancers and therefore to a longer survival (or a longer lead time). However, before studying the possible effect of surveillance and other prognostic factors (which was not the aim of this study), we should focus on the correct measurement of survival, which is our research objective.

To achieve this, we had to face many complex methodological challenges: first, we had to fix the zero reference time (the time from when we started the follow-up); second, a person can die only once thus the background death rate is confounded in the follow up information after the diagnosis of the second primary, therefore it is crucial to use models able to suitably describe a situation of competing risks; third, in order to make inferences, for each model we had to define the correct expected survival based on the appropriate comparison group.

We focused our attention on the following questions:

A. Does the survival probability of a patient with a second primary tumour differ from those with only first type of tumour?

B. Does the survival probability of a patient with a second primary tumour differ from those with only second type of tumour?

If a difference in survival is found in some of the previous situations, a third more fundamental question arises.

C. Are the probabilities of surviving two subsequent primary tumours independent?

Studying survival probabilities in terms of the underlying hazard of death, the question can be rephrased as follow:

Is the mortality rate after a second tumour simply the sum of the two intensities (additivity hypothesis), or the way the mortality rates act follows a different functional law?

This paper aims at answering these questions for women with breast cancer and a subsequent primary tumour, paying particular attention to the conditional survival probability due to the time elapsed between the two malignancies.

## Methods

### Statistical analysis

To correctly defining the probability of surviving conditional to be alive up to the occurrence of a second tumour, we started by writing questions A, B and C as hypothesis in term of mortality hazard. We defined:

*λ*_*A*_(*t*): mortality rate for the population with two tumours at time *t *from the occurrence of the first tumour;

*λ*_*B*_(*t*): mortality rate for the population with two tumours at time *t *from the occurrence of the second tumour;

*λ*_*C*, *α*_(*t*): mortality rate at time *t *from the occurrence of the second tumour for the population with a second tumour given that they already survived a time interval *α*.

We can break these down as

*λ*_*A*_(*t*) = *λ*_1|0_(*t*) + *λ*_1|2_(*t*) + *λ*_0_

*λ*_*B*_(*t*) = *λ*_2|0_(*t*) + *λ*_2|1_(*t*) + *λ*_0_

where, for *i *= 1, 2, *λ*_*i*|0 _is the specific mortality rate at time *t *from the occurrence of tumour i for the population with only that tumour, and *λ*_0 _is the general mortality.

We assumed that *λ*_1|0_, *λ*_2|0 _and *λ*_0 _were known, by previous studies on mortality and survival in population with the first type of tumour only, with the second type of tumour only, and in the general population, respectively.

We observed that *λ*_1|2_(*t*) was the possible difference in mortality rate in patients with a tumour of type 1 followed by a tumour of type 2 with respect to that of patients with a tumour of type 1 only, measured from the occurrence of tumour 1; *λ*_2|1_(*t*) was the possible difference in mortality rate in patients with a tumour of type 1 followed by a tumour of type 2 with respect to that of patients with a tumour of type 2 only, measured from the occurrence of tumour 2.

Questions A, B, and C can be written as follows:

$$\begin{array}{ll} A. & \lambda_{1|2}\overset{?}{\underset{}{=}}0\\ B. & \lambda_{2|1}\overset{?}{\underset{}{=}}0\\ C. & \lambda_{C,\alpha }(t)\overset{?}{\underset{}{=}}\lambda_{1|0}(t+\alpha )+\lambda_{2|0}(t)+\lambda_{0} \end{array}$$

Occurrence probabilities conditioned to different events (occurrence of a second cancer, death) in each time interval can be estimated with the Aalen-Johansen [8] (**AJ**) method in the framework of a Markov process, as described later. Once we obtained these conditional probabilities, we calculated the number of expected deaths by sex, age, calendar period and follow-up time, under the different hypotheses A, B and C. From a practical point of view, we calculated the expected deaths in a similar way to that used to calculate the denominator of relative survival [9]. For example, in the case of a woman diagnosed with breast cancer at 62 who developed a rectal cancer after two years and survived for an additional period of five years, we associated an expected probability of dying with a breast cancer, occurred at the same age, for the two years elapsed with that cancer only. Subsequently, we associated an expected probability of dying with breast and/or with rectal cancer for the following years, taking into consideration the ageing of the patient (i.e. using the annual probability of dying according to the age of the patient, from age 64 to age 69). The way the calculation of the expected number of death (or the expected probability of dying) for the conjoint period when both tumours are present is performed depends on which one of the three hypotheses we are testing. If we consider hypothesis A, we do not add the probability of dying with a colon-rectum cancer. If we test hypothesis B, we do not add the probability of dying associated to a breast cancer for the first period. Finally, if we test hypothesis C (additive hypothesis), we sum the two underlying mortality hazards during the second period. Expected probabilities were derived from analyses of the cohort of patients with only one incident cancer included in the cancer registry's data.

For the interested reader, we now explain in details how we calculated expected probabilities. Since different states are concerning, we resorted to the theory of Markov models [8]. In a Markov process individuals can belong to a finite set of states and move to one state to some others with a probability, possibly depending on time. The main hypothesis is that the probability of moving from state *i *to state *j *at time *t *depends on *i*, *j *and *t *only, and not on the previous history of the individual.

We constructed a simple model with three states

1 first tumour

2 second tumour

3 death after a first (but not a second) tumour

where 2, and 3 are absorbing states and the possible moves are: 1 → 2, 1 → 3.

Since our data showed right censoring, transition probabilities *P*_*ij*_(*s*, *t*) from state *i *to state *j*, in the time interval (*s*, *t*) were calculated using Aalen-Johansen (**AJ**) estimators [8].

The procedure we adopted included age standardisation, and precisely:

• For each age class *k *we calculated the **AJ **estimator *P*_*ijk*_(*s*, *t*). We let *N*_*k *_be the number of subjects in class *k *at time 0 and we set a weight $W_{k}=\frac{N_{k}}{N}$, where *N *equals the sum of the *N*_*k*_'s.

• We defined the standardised estimator as: $P_{12}^{stand}(s,t)=\sum_{k}W_{k}\cdot P_{12k}(s,t)$

• It is reasonable to assume that weights are deterministic (fixed) variables; under this assumption we have:

$$var(P_{12}^{stand}(s,t))=\sum_{k}W_{k}^{2}\cdot var(P_{12k}(s,t)).$$

Then, from probabilities previously calculated with **AJ **estimators, it was possible to compare observed mortality with mortality expected in the hypothesis of no interaction between the two tumours; that is the mortality intensities due if the two tumours were independent. As a consequence, the number of expected deaths is the sum of the deaths due to mortality for both tumours acting separately. We calculated the number of expected deaths considering for each patient *j *the time of occurrence of the first primary malignancy *T*_1*j*_, time of occurrence of the second primary malignancy *T*_2*j*_, and, most important, the time interval between the occurrence of the two tumours *α*_*j *_= *T*_2*j *_- *T*_1*j*_. Each patient, after a time interval *t*_2 _since the inception of the second tumour, has a probability *p*_2*j*_(*t*_2_) of dying for the second tumour or general mortality equal to that of the general population of patients with only that type of tumour, according to her/his age, sex, calendar period of diagnosis and follow-up time. In addition, that patient has a probability *p*_1*j*_(*t*_2 _+ *α*_*j*_) of dying at the (*t*_2 _+ *α*_*j*_)- time interval for the first tumour or general mortality again equal to that of the general population of patients with that type of tumour only, according to her/his age, sex, calendar period of diagnosis and follow-up time.

We set ${\hat{p}}_{2j}$(*t*_2_) = *p*_2*j*_(*t*_2_)·(1 - *p*_0*j*_) where *p*_0*j *_is the general mortality of the subject *j *according to her/his age, sex, calendar period of diagnosis, taken from the life tables of the general population. Thus, we can say that ${\hat{p}}_{2j}$(*t*_2_) is the specific mortality for the second tumour.

Therefore, within the cohort of patients with two malignancies, at the *t*_2 _time interval since the second tumour, in the hypothesis of no interaction between the mortality forces of the two tumours, we expect the following number of deaths:

$$\sum_{j}\left\{p_{1j}(t_{2}+\alpha_{j})+[1-p_{1j}(t_{2}+\alpha_{j})]\cdot {\hat{p}}_{2j}(t_{2})\right\};$$

where the probability of dying for the second tumour ${\hat{p}}_{2j}$(*t*_2_) is corrected by the probability of surviving from the first tumour and general mortality 1 - *p*_1*j*_(*t*_2 _+ *α*_*j*_).

Since the output of these calculations was the number of expected deaths, we consequentially compared it with the observed number in a ratio similar to the well known *Standardised Mortality Ratio*:

$$SMR_{AJ}=\frac{\text{Number of observed deaths at time }t}{\text{Number of expected deaths at time }t}.$$

We used the term *SMR*_*AJ *_because it was quite similar to the standard term "SMR"in the sense that it was that ratio of observed to expected deaths; the expected deaths were calculated as a sum over age groups; and finally, it was similar to the indirect method of age standardisation since, as standard, we applied the mortality rates of the cohort of patients with only one tumour.

### Patients

We selected all incident breast cancer cases recorded by the Piedmont Cancer Registry in the resident women of Turin from 1985 to 1998. This cohort of patients was followed up for four years until the end of 2002, both for what concerns life status or development of a subsequent tumour (excluding skin carcinoma). Life status of women who emigrated outside the resident population observed by the Piedmont Cancer registry was ascertained with an active follow-up at the municipality rosters of the new residency. In this analysis we considered women with cancer of corpus uteri or cancer of colon-rectum as second primary tumour, since we observed a consistent number of cases (91 for colon-rectum and 62 for corpus uteri) for making reasonable stable estimates. In addition, we analysed all types of second primary cancers (escept skin carcinomas) including corpus uteri and colon-rectum. For age standardisation, we introduced age at diagnosis in five broad classes: 0 – 44, 45 – 54, 55 – 64, 65 – 74, 75+. Age standardisation for the unconditional survival estimates were calculated using those standards proposed by Corazziari et colleagues [10] for comparisons in international studies.

We calculated *λ*_1|0 _from our cohort of 8234 women with breast cancer only. Mortality for the second type of tumour (*λ*_2|0_) was calculated from 1443 women with corpus uteri cancer only, and from 4050 women with colon-rectum cancer only. In the case of mortality for all cancers *λ*_2|0 _was calculated in two ways: including breast cancers (28737 women), and excluding breast cancers (20082 women). We also used overall mortality including breast cancer, as a reference for comparing available published statistics that usually do not make exclusions for specific type of cancers. Life tables for the general mortality were from the Statistics Office of Turin for the period 1985–2002.

## Results

We identified 9233 women with breast cancer in Turin from 1985 to 1998, 563 cases were excluded as they were identified from Death Certificate Only (DCO) or synchronous primary tumours (same day of diagnosis for the two tumours) or they were patients with more than two tumours, leaving 8670 cases for analysis. From this cohort, 436 second (metachronous) primary tumours developed during the prolonged follow-up period (1985–2002). The most frequent types of subsequent cancers were colon-rectum (91 patients) and corpus of uteri (62 patients) (Table 1). The median interval time between the two tumours was five years, but with an highly positive skewed distribution. The completeness of clinical documentation was rather high, considering that registries work on a population basis, with a 94.5% of microscopic confirmation for first tumours that reached 99.3% for second tumours.

**Table 1:** Distribution of subsequent primary malignancies and deaths among a cohort of women with breast cancer in Turin from 1985 to 1998 (follow-up 2002)

**Second cancer**	**Number of cases**	**Observed deaths**
Head & Neck	16	11

Stomach	31	28

Colon-Rectum	91	57

Liver & Gallbladder	20	19

Pancreas	25	23

Lung & Pleura	34	32

Melanoma of skin	15	2

Cervix Uteri	10	5

Corpus Uteri	62	23

Ovary	29	20

Bladder	14	7

Kidney	12	5

Brain & CNS	5	5

Thyroid Gland	14	5

NHL	24	16

Leukaemia	13	9

Others	21	18

**Total**	**436**	**285**

At the end of the study period (2002), we observed 285 (65.4%) deaths among women with a second tumour, distributed as in table 1, and 3931 (47.7%) among women with breast cancer only.

Using a traditional approach, starting from the diagnosis of the first tumour (breast cancer), we had, at 1, 5 and 10 years respectively, an age standardised survival of 96.1%, 74.3% and 43.6% for the 436 women with two cancers, while it was 94.0%, 72.9% and 56.9% for women with breast cancer only (Table 2). On the contrary, survival of women with one tumour only (all site including breast cancer) was worse: 69.5%, 47.5%, and 37.1% after 1, 5 and 10 years of follow-up. Also survival of women with colon-rectum or corpus uteri cancer only was worse than that of women with a subsequent cancer, when starting follow-up since diagnosis of breast cancer. Their higher survival is due to the fact that patients with multiple cancers had survived an extra amount of time (a median of five years) before developing the subsequent cancer in comparison with patients with a single cancer only. A less biased and more appropriate comparison can be obtained moving the zero time point to the date of diagnosis of the second tumour. In this case, the survival of patients with breast cancer and a subsequent tumour was rapidly decreasing since the date of diagnosis of the second tumour, and comparable to that of patients with only one tumour excluding breast cancer. Also survival of patients with breast and colon-rectum cancer was rapidly decreasing after the first year, when it was still comparable, and lower (20.8% versus 32.9%), although not statistically significantly, than that of patients with colon-rectum cancer only. Survival of patients with breast and corpus cancers was generally quite high, with 70.6% of women still alive after 10 years since the breast cancer and 44.2% alive after 10 years since the corpus uteri cancer diagnosis. However, patients with corpus cancer only showed a higher survival at 10 years (56.6%).

**Table 2:** Age standardised observed survival (%) according to various traditional unconditional approaches – at 1, 5 and 10 years of follow-up in women with two cancers (since the diagnosis of the first or second tumour) compared with women with one cancer only.

**PATIENTS GROUPS**	number of patients	1 year since diagnosis	5 years since diagnosis	10 years since diagnosis
One primary cancer only (all tumours excluding breast cancer) (95% C.L.)	20082	58.9 (58.3–59.2)	36.5 (34.9–37.1)	28.5 (27.7–29.3)

One primary cancer only (all tumours including breast cancer) (95% C.L.)	28316	69.5 (68.9–71.1)	47.5 (46.9–48.1)	37.1 (36.3–37.9)

Breast cancer only (95% C.L.)	8234	94.0 (93.4–94.6)	72.9 (70.0–73.9)	56.9 (55.5–58.3)

Colon-Rectum cancer only (95% C.L.)	4050	68.4 (66.8–70.0)	42.9 (41.1–44.7)	32.9 (30.7–35.1)

Corpus Uteri cancer only (95% C.L.)	1443	87.8 (86.0–89.6)	69.0 (51.1–82.5)	56.6 (15.9–76.7)

Breast cancer with second primary cancer, f.u. starting from breast cancer diagnosis (95% C.L.)	436	96.1 (93.8–97.5)	74.3 (69.5–78.5)	43.6 (36.6–50.9)

Breast cancer with second primary cancer, f.u. starting from second primary cancer diagnosis (95% C.L.)	436	61.7 (57.0–66.1)	37.8 (29.5–46.8)	24.6 (10.8–46.8)

Breast cancer with subsequent Colon-Rectum cancer with f.u. starting from breast cancer diagnosis (95% C.L.)	91	97.8 (92.3–99.4)	72.4 (61.7–81.1)	43.7 (28.7–59.9)

Breast cancer with subsequent Colon-Rectum cancer with f.u. starting from Colon- Rectum cancer diagnosis (95% C.L.)	91	70.3 (60.3–78.7)	38.9 (21.9–58.9)	20.8 (2.9–69.2)

Breast cancer with subsequent Corpus Uteri cancer with f.u. starting from breast cancer diagnosis (95% C.L.)	62	100.0 -	91.9 (82.2–96.6)	70.6 (53.7–83.2)

Breast with subsequent Corpus Uteri Cancer with f.u. starting from Corpus Uteri cancer diagnosis (95% C.L.)	62	88.7 (78.5–94.4)	68.9 (51.1–82.5)	44.2 (15.9–76.7)

**Figure 1:**
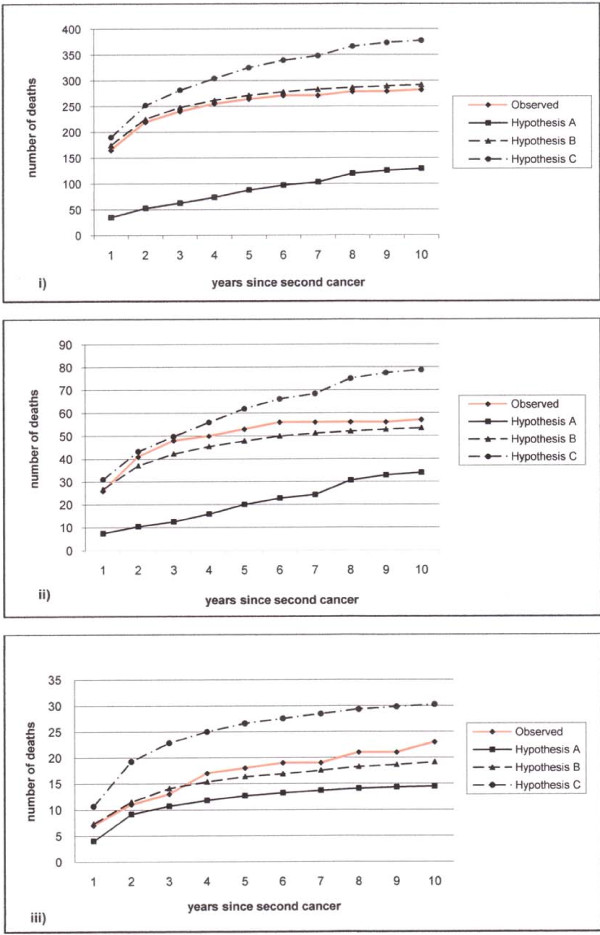
**Cumulative number of deaths following different hypotheses for women with a second cancer after breast cancer**: (i) all tumours (ii) colorectal cancer (iii) corpus uteri cancer.

In table 3 we compared the number of observed deaths with the expected deaths under the three hypotheses A, B and C. We calculated the number of expected deaths taking into account the probabilities of surviving the first tumour, developing a second tumour and subsequently dying. We presented the expected deaths broken down according to the three different probabilities. The expected numbers of deaths under the first hypothesis (A) were definitively below the observed numbers since they did not take into account the probability of dying because of the second tumour. On the other hand, the expected number of deaths from the unconditional probabilities of dying for the second tumours (hypothesis B) were overestimated (291.8) in the case of all cancers and slightly underestimated in the case of colon-rectum (53.45) and corpus uteri cancers (19.16). The expected numbers of deaths according to the probabilities of dying because of the second tumour, given that the patients survived the first, were 248.30 for all cancers, 44.76 for colon-rectum cancer and 15.81 for corpus uteri. Summing up the latter figures with the expected numbers for hypothesis A, we obtained the expected number of deaths according to the additivity hypothesis C. Considering the ratio between observed and expected numbers (*SMR*_*AJ*_), the additivity hypothesis is not confirmed for all cancers (*SMR*_*AJ *_0.75 95% CL 0.66–0.84), or for colon-rectal cancer (*SMR*_*AJ *_0.72 95% CL 0.55–0.94) while in the case of corpus uteri cancer we cannot reject the hypothesis that mortality rates are additive. The differences between the three models and observed deaths is visualised in Figure 1 where cumulative numbers of deaths are displayed over years of follow-up. It can be seen that in all graphs hypothesis C (additivity) tends to overestimate the actual observed trend, hypothesis A strongly underestimates it, while hypothesis B is the closest to observed data, especially during the first time (years) of follow-up.

**Table 3:** Observed and expected number of deaths, according to various hypotheses, in women with breast cancer and with a subsequent primary tumour after 10 years from the reference time.

	**Conditional probability**	**Subsequent primary tumour**		
		**All cancers**	**Colon-Rectum**	**Corpus Uteri**

observed number of deaths		285	57	23

expected number of deaths from the first (breast) cancer - Hypothesis A	*p*_1_(*t *+ *αj*)	129.22	34.01	14.45

*SMR*_*AJ *_– **Hypothesis A** (95% CL)		**2.21** (1.94 – 2.45)	**1.68** (1.27 – 2.17)	**1.59** (1.01 – 2.39)

expected number of deaths from the second cancer - Hypothesis B	*p*_2_(*t*)	291.8	53.45	19.16

*SMR*_*AJ *_– **Hypothesis B** (95% CL)		**0.98** (0.87 – 1.10)	**1.07** (0.81 – 1.38)	**1.20** (0.76 – 1.80)

conditional expected number of deaths from the second cancer	${\hat{p}}_{2}$(*t*)·(1 - *p*_1_(*t *+ *αj*))	248.30	44.76	15.81

expected deaths based on conditional probabilities - Hypothesis C (additive)	*p*_1_(*t *+ *αj*)+ + ${\hat{p}}_{2}$(*t*)·(1 - *p*_1_(*t *+ *αj*))	377.5	78.77	30.26

*SMR*_*AJ *_– **Hypothesis C** (95% CL)		**0.75** (0.66 – 0.84)	**0.72** (0.55 – 0.94)	**0.76** (0.48 – 1.14)

Expected numbers were calculated stratifying by age classes and calendar period (hypotheses A and B) and allowing for general mortality in women (hypothesis C). See Methods for definition of probabilities *p*_*i *_and of *SMR*_*AJ*_.

## Discussion

The dramatic improvement of cancer survival during the last decades in Western countries brought with it a new health threat: the development of second primary cancers in survivors. An editorial in CEBP of David Alberts clearly stated that 'Second cancers are killing us! ' [11]. However, in spite of the fact that several studies on the multiple primary cancer risk were undertaken [12], the rate at which first and second, or higher-order cancers are killing us remains neglected. In clinical studies, when reliable information are available it is often possible to understand if the pathological conditions linked to a specific cancer affected the patient survival and to which extent. However, at a population based level this is often not feasible due to the lack of clinical information or cause of death. Even when cancer-specific causes of death are available, they are subject to various degrees of misclassification, hindering the possibility of a reliable estimate of cancer specific survival. In the main population based statistics on cancer survival worldwide available (Eurocare [3] and SEER [4]) subsequent cancers were excluded: only the first occurring cancer was analysed, or all the subjects with multiple cancers were deleted from analysis. Although, this strategy has recently undergone through a rethinking [5,6], it was supposed to allow for more comparable results across registries with different back up information, and therefore with a different possibility in identifying those cancers that occurred in prevalent cases. However, we believe that the problem deserves more attention also from its implication in the management and care of such patients. Indeed, a wider availability of effective cancer treatments has prolonged patient survival, so increasing the possibility of developing another cancer. Studying the occurrence of multiple tumours and their association, it emerged as the higher susceptibility to subsequent malignancies can possibly be due to unfavorable genetic pattern or common exogenous risk factors [13,14]. Multiple cancer survival is also a stimulating topic of study, but received less attention. Recently, an analysis of the SEER data on multiple tumours following breast cancer [15] showed that survival of women 20–29 years old at time of breast cancer diagnosis had a worse 10-year survival, compared with women with breast cancer only, while there were no differences in the 5-year survival. However, in that analysis the time elapsed until the second cancer occurrence was not taken into account.

Before investigating the reasons influencing survival for patients with multiple tumours, we, indeed, believe that it is essential to have a correct measurement of survival that takes into account the effect of conditional probabilities of surviving given the different timing of primary cancers occurrences. We proposed a method that assigns the correct number of expected events according to the different components of mortality due to each type of cancer. The proposed method is useful only in correctly stating the prediction of mortality probabilities while cannot explain the causes of the different mortality probabilities.

The expected number of deaths was calculated taking into account the exact time spent at risk of dying for one or another cancer by age classes and calendar period, using conditional probabilities estimated by the **AJ **estimator from a simple one-way Markov process with two absorbing states. Such approach was recommended since it allowed a better control of probabilities of events arising from different states. In the model referring to hypothesis A, we calculated the expected number of deaths due to the first occurring cancer starting since its time of occurrence. This model is similar to model 2 proposed by Heinävaara and colleagues [7] in the absence of cancer specific cause of death. We wrote the model's parameters in terms of risk excess (hazard rate), rather than estimating the specific mortality rates. While survival of patients with a second primary tumour was comparable or higher with that of those patients with breast cancer only during the first years, it was rapidly declining at a higher rate than the reference group after five years of follow-up. This effect was explained by the fact those patients had survived an extra amount of time (a median of five years) before developing the subsequent cancer. Indeed, results from hypothesis A showed an increased cumulative mortality only at ten years for women with two cancers when compared to those with breast cancer only, as found in the study of Raymond and Hogue [15].

The second model (hypothesis B) was built with the same structure as model A, calculating the expected number of deaths due to the second occurring cancer starting since its time of occurrence. However, the change in the baseline population and the shift in the time zero reference made the hazard rates not comparable. Indeed, for a proper comparison with those patients with the second type of cancer only, we set the starting time at the diagnosis of the second cancer. In this case, the survival was comparable at 1 and 5 years of follow-up, than that of patients with one type of cancer only, while it was slightly shorter at 10 years. In summary, results from hypothesis B showed no extra mortality compared to patients with only one cancer of the same type, and observed and expected number of deaths closely get on during the years of observation.

We then addressed the question of evaluating the eventual extra mortality due to the combination of effects of the two primary neoplasms, checking the hypothesis if the mortality of women with two cancers was due to the sum of the baseline mortality rates of breast and other cancers (additivity hypothesis C). It clearly emerged how observed cumulative mortality was lower than expected under the additivity assumption, with a statistically significant difference in the case of all cancers and colon-rectum after 10 years of follow-up. The agreement of a specific model to observed data was therefore useful for having further hints of the underlying mechanisms. In our study, the less than expected results can be explained by the fact that the second cancer can have a less advanced stage and therefore a better prognosis, since a subsequent cancer is usually diagnosed because of a deeper clinical surveillance due to the first cancer. It is clear that women with breast cancer and a subsequent cancer survive less than women with breast cancer only, but their survival is not always decreased simply as it would be if the forces of mortality work together in an additive way.

The study has some possible limitations. First of all, the method of correction is based on observed rates (mortality rates measured in the cohort of patients with only one tumour) that, when based on small numbers, can be unstable. Then, this method, being inherently non-parametric, does not give information on the underlying incidence/mortality competing laws. In calculating expected number of deaths a possible bias could have been introduced, depending on the numbers of patients who emigrated outside the Cancer Registry's area. In this case, information on life status were still available and collected, but we did not know if the patient had developed a subsequent cancer when resident in another area. During the study period, we observed about 8% of women who emigrated among those classified with breast cancer only. Their median time of emigration was 6.5 years since the breast cancer diagnosis. As a consequence, considering that the median time for developing a second primary cancer was about 5 years, the detection bias should be very limited. Finally, the method was presently tested only on a limited set of data: patients with breast cancer as a first primary tumour. As few studies are still available on this topic, more research is needed, with larger samples and including clinical data (e.g. stage at presentation, hormone receptor status), therapies (e.g. tamoxifen), information on follow-up circumstances, and modality of diagnosis. In conclusion, we showed that the presented approach for calculating conditional probabilities was correct when dealing with situations, as with multiple tumours, where competing causes of death can bias the results of survival probabilities. We also pointed out how shifted reference times can be considered in correctly comparing survival. In addition, departure from the expected additive model can give hints towards which direction to further investigate.

## Authors' contributions

SR conceived the idea for the study. SR, FR and LT planned and designed the research. FR developed the statistical models. LT revised the mathematical foundations of the proposed model. FR and LT analysed the data. SR and RZ wrote the first draft of the manuscript. RZ coordinated this project. All authors edited and approved the final version of the manuscript. The corresponding author has final responsibility to submit for publication.
